# Multi-Dimensional Molecular Regulation of Trichome Development in *Arabidopsis* and Cotton

**DOI:** 10.3389/fpls.2022.892381

**Published:** 2022-04-07

**Authors:** Yanan Wang, Qi Zhou, Zhigang Meng, Muhammad Ali Abid, Yuan Wang, Yunxiao Wei, Sandui Guo, Rui Zhang, Chengzhen Liang

**Affiliations:** Biotechnology Research Institute, Chinese Academy of Agricultural Sciences, Beijing, China

**Keywords:** trichome, *Arabidopsis*, cotton, fiber, multi-dimensional regulation

## Abstract

Plant trichomes are specialized epidermal cells that are widely distributed on plant aerial tissues. The initiation and progression of trichomes are controlled in a coordinated sequence of multiple molecular events. During the past decade, major breakthroughs in the molecular understanding of trichome development were achieved through the characterization of various trichomes defective mutants and trichome-associated genes, which revealed a highly complex molecular regulatory network underlying plant trichome development. This review focuses on the recent millstone in plant trichomes research obtained using genetic and molecular studies, as well as ‘omics’ analyses in model plant *Arabidopsis* and fiber crop cotton. In particular, we discuss the latest understanding and insights into the underlying molecular mechanisms of trichomes formation at multiple dimensions, including at the chromatin, transcriptional, post-transcriptional, and post-translational levels. We summarize that the integration of multi-dimensional trichome-associated genes will enable us to systematically understand the molecular regulation network that landscapes the development of the plant trichomes. These advances will enable us to address the unresolved questions regarding the molecular crosstalk that coordinate concurrent and ordered the changes in cotton fiber initiation and progression, together with their possible implications for genetic improvement of cotton fiber.

## Introduction

Plant trichomes are the unicellular or multicellular appendages originating from epidermal cells and are widely distributed on the surface of different organs of plants, i.e., stems, leaves, petioles, flowers, and seed coats (Hülskamp et al., 1994; Pattanaik et al., 2014). Trichomes on vegetative organs are physical barriers that protect against ultraviolet (UV) radiation, excessive transpiration, and insect herbivory (Serna and Martin, 2006; Wang et al., 2019b). Seed coat trichomes primarily increase seed dispersal, but are also significant resources in the textile industry (e.g., cotton fibers; Wang et al., 2019b). Trichome development is initiated by a variety of external environmental factors and endogenous developmental signals (Serna and Martin, 2006; Qi et al., 2011). Environmental factors include wounding and insect attack, which are associated with trichome phenotypes. Variations in trichome density are likely a result of adaptation to different environments (Arteaga et al., 2021). Endogenous developmental signals include phytohormone signals. Gibberellic acid (GA) and jasmonic acid (JA) alter trichome development by crosstalk with transcription factors (Yan et al., 2017). In addition, cytokinin (CK), salicylic acid (SA), and ethylene also affect trichome development (Maes et al., 2008; Matías-Hernández et al., 2015; Yu et al., 2022).

**Figure 1:**
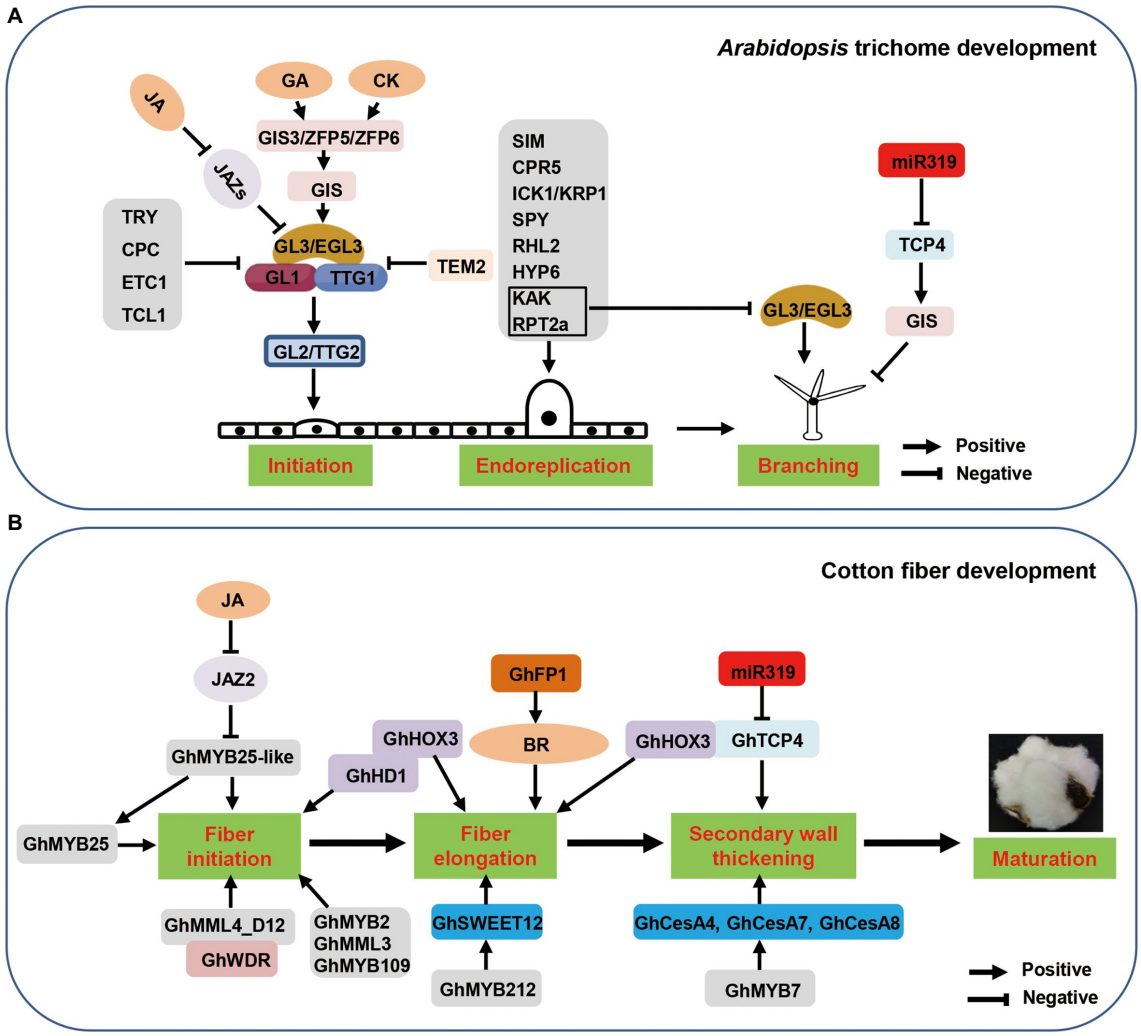
Core regulators controlling *Arabidopsis* trichome development and cotton fiber development. **(A)** Key regulators are involved in *Arabidopsis* trichome development. GL1-GL3/EGL3-TTG1 is the core complex to regulate *Arabidopsis* trichome initiation, which is needed for the activation of GL2/TTG2. C2H2 zinc finger proteins GIS3, ZFP5, and ZFP6 that regulate trichome initiation through GA and CK signalling (Sun et al., 2015). *GL2* function partially redundant with *TTG2*, downstream of the GL1-GL3/EGL3-TTG1 in trichome initiation. Single-repeat MYBs TRY, CPC, ETC1, and TCL1 prevent the formation of GL1-GL3/EGL3-TTG1 complex. TEM2 significantly represses the expression GL1 and EGL3 to inhibit trichome development. Some regulators affect trichome development by endoreduplication pathway. In addition, KAK and RPT2a inhibit the expression of *GL3/EGL3* causing reduced trichome branching. In addition to regulating trichome initiation, GL3/EGL3 is also involved in trichome branching. TCP4 suppresses trichome branching by direct transcriptional activation of *GIS* (Wang et al., 2019b). **(B)** Key regulators are involved in cotton fiber development. MYB, bHLH, and HD-ZIP TFs are important regulators in cotton fiber development. GhMYB25 and GhMYB25-like have been identified as regulators of fiber initiation, and GhJAZ2 negatively regulates fiber initiation by interacting with GhMYB25-like. GhMML4 is known to regulate the fiber development by binding with GhWDR. GhHD1 regulates fiber initiation, while GhHOX3 regulates fiber elongation. GhHOX3 interacts with GhHD1, enhancing transcriptional activity of GhHOX3. In fiber elongation stage, GhMYB212 directly controls expression of sucrose transporter gene *GhSWEET12* (Wang et al., 2019b). GhFP1 directly binds to *GhDWF4* and *GhCPD* promoters to activate BR biosynthesis and signaling to regulate fiber elongation. GhMYB7 directly regulates fiber cellulose synthesis by binding to three different cis-elements in the *GhCesA4*, *GhCesA7*, and *GhCesA8* promoters. GhTCP4 maintains the balance between cotton fiber cell elongation and cell wall synthesis by interacting with GhHOX3.

*Arabidopsis thaliana*, a model plant, has trichomes that are typically unicellular, non-glandular, and have two to three branches (Szymanski et al., 2000; Grebe, 2012). Similarly, cotton fibers are also composed of non-glandular (but non-branched) single cells (Wang et al., 2019b). In *Arabidopsis*, trichome development generally involves initiation followed by four rounds of endoreplication and branching (Hülskamp et al., 1994; Yang and Ye, 2013; Figure 1A). Many studies have uncovered that trichome initiation begins when epidermal cells collect signals from neighboring cells and subsequently undergo cell differentiation regulated by evolutionarily conserved transcription factors that are involved in patterning processes and trichome morphogenesis. Cotton fiber development is also divided into four major sequential and overlapping developmental stages: fiber initiation, fiber elongation (primary cell wall synthesis), cell wall thickening (secondary cell wall deposition), and fiber maturation (Figure 1B). Currently, molecular mechanisms of fiber development (especially initiation and elongation) are widely studied, and some key genes have been characterized. Due to their unique cell structures, trichomes serve as an excellent model system to study all aspects of plant differentiation at the single cell level including cell fate differentiation and morphogenesis (Balkunde et al., 2010; Yang and Ye, 2013).

Cotton fiber and *Arabidopsis* trichome initiation share some similarities but have a different mode of molecular regulation (Tian and Zhang, 2021; Wang et al., 2021a). Several key genes involved in *Arabidopsis* trichome initiation have been identified that form a transcriptional network involving three major groups of transcription factors: R2R3-type MYB-basic helix–loop–helix (bHLH)-WD40 repeat (WDR) protein (Payne et al., 2000; Serna and Martin, 2006; Grebe, 2012; Pattanaik et al., 2014; Tian and Zhang, 2021). These regulatory proteins form a trimeric activator complex (MBW complex) that positively regulates trichome initiation by activating expression of *GLABRA2* (*GL2*; Arteaga et al., 2021). Genetic and molecular evidences have demonstrated that the MBW complex interacts with DELLA and JAZ proteins and mediates GA and JA signaling to control trichome initiation (Qi et al., 2014). In addition, mutations in MBW complex genes lead to smaller and less branched trichomes (Payne et al., 2000). Single repeat R3-MYB proteins have been reported to repress trichome initiation by interfering the function of the MBW complex (Schellmann et al., 2002; Wang et al., 2007; Wester et al., 2009; Wang and Chen, 2014), which results in the repression of *GL2* (Serna and Martin, 2006; Ishida et al., 2008; Wang and Chen, 2014; Doroshkov et al., 2019). In cotton, some genes that control fiber development have been identified as homologues of *Arabidopsis* trichome regulators (Walford et al., 2012; Guan et al., 2014a; Table 1). Ectopic expression of some of these cotton homologues alters trichome development in *Arabidopsis* (Wang et al., 2004; Zhang et al., 2010; Guan et al., 2014a). However, a cotton (*Gossypium hirsutum*) MIXTA-like transcription factor, GhMML4_D12, is known to regulate the fiber development by binding with GhWDR but not with a bHLH protein (Tian and Zhang, 2021). Moreover, several spontaneous “lintless” or “naked” cotton mutants have normal leaf and stem trichomes (Ruan, 2005; Tian and Zhang, 2021; Wang et al., 2021a). This evidence suggests that cotton fiber initiation differs from trichome initiation on vegetative organs.

**Table 1:** Functional homologous genes related to *Arabidopsis* trichome and cotton fiber patterning.

Genes	Regulation	References	Genes	Regulation	References
*AtGL1*	Initiation	Oppenheimer et al., 1991	*GhMYB2*	Initiation	Guan et al., 2014a
*AtGL2*	Initiation	Rerie et al., 1994	*GaHOX1*	Initiation	Guan et al., 2008
*AtGL3*	Initiation and branching	Payne et al., 2000	*GhDEL65*	Initiation and elongation	Shangguan et al., 2016
*AtPDF2*	Epidermal cell differentiation	Abe et al., 2003	*GbML1*	Initiation	Zhang et al., 2010
*AtTTG1*	Initiation	Walker et al., 1999	*GhTTG1/ GhTTG3*	Initiation	Humphries et al., 2005
*AtHUB2*	NR		*GhHUB2*	Elongation and SCW deposition	Feng et al., 2018

*NR: No report*.

**Figure 2:**
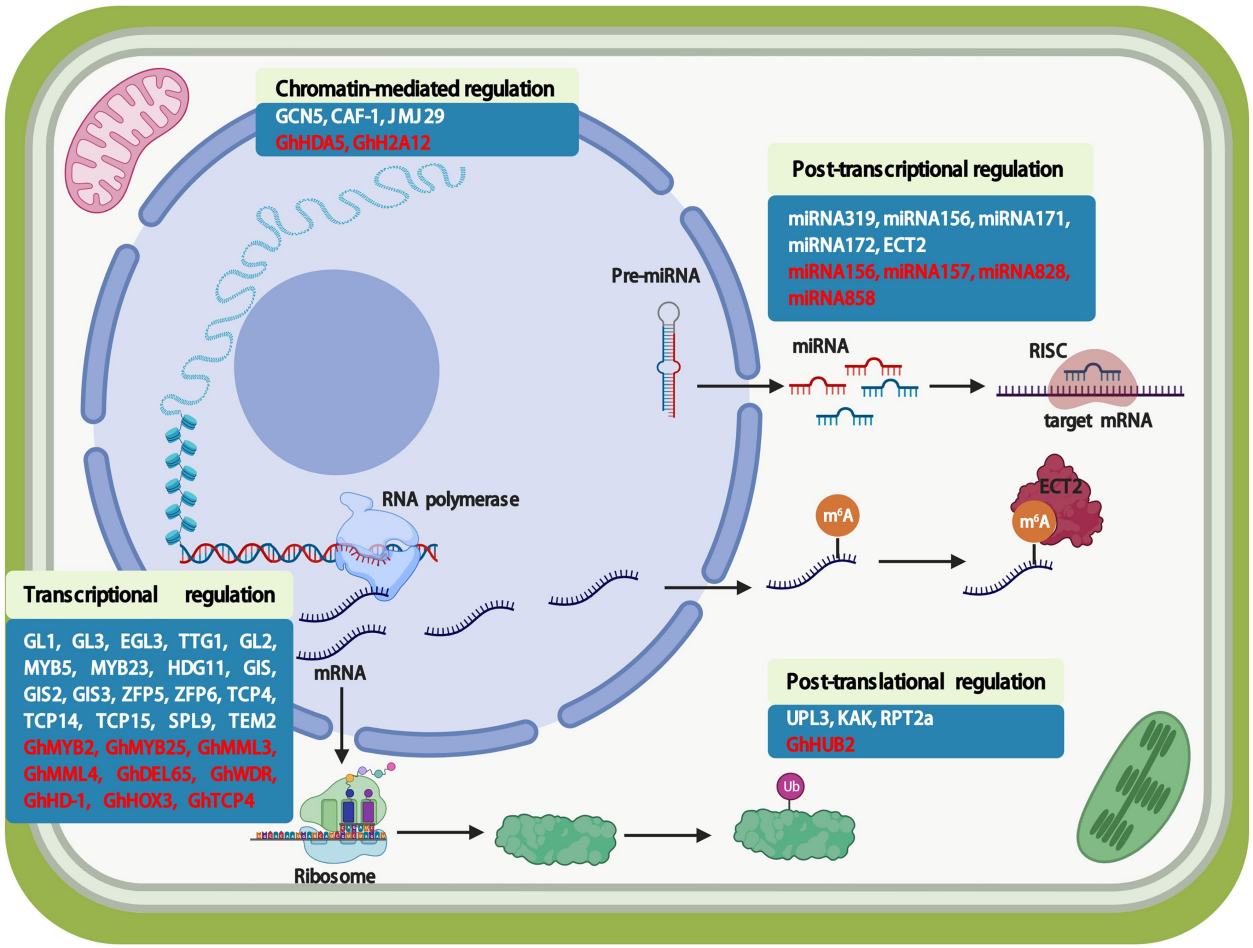
Multi-dimensional regulation of *Arabidopsis* trichome and cotton fiber development. The control of trichome and fiber development involves multi-dimensional regulation. Here shows chromatin-mediated, transcriptional, post-transcriptional, and post-translational regulation modes. Chromatin-mediated regulation of trichome and fiber development, including histone modification and maintenance of chromatin states. Multiple transcription factors are involved in trichome and fiber development. The post-transcriptional regulation of trichome and fiber development is mainly caused by miRNA. Post-translational modifications, such as ubiquitination, is important in regulating trichome and fiber development. White words represent the genes are involved in trichome-related genes in *Arabidopsis*; Red words represents the genes are involved in cotton fiber development. Pre-miRNA: premature microRNA; m^6^A: N^6^-methyladenosine; RISC: RNA-induced silencing complex; Ub: ubiquitin.

As described above, the regulatory network of trichome development is rigorous and complex. Thus, it is necessary to dissect molecular mechanisms of trichome development in multiple dimensions in order to understand the genetic mechanisms involved. In this review, we focus on multi-dimensional regulatory modes in *Arabidopsis* and cotton, describing the latest results in research related to chromatin-mediated, transcriptional, post-transcriptional, and post-translational regulation of trichome development (Figure 2). These data provide valuable insight into the regulatory network of trichome development, ultimately accelerating understanding of the molecular mechanisms of cotton fiber development.

## Transcriptional Regulation of Trichome Development

Transcription factors (TFs) are the main drivers of evolution and domestication because they have the potential to fine-tune traits and improve crops (Schilling et al., 2019). Previous studies have clarified the identity of some TFs involved in trichome improvement (Oppenheimer et al., 1991; Rerie et al., 1994; Morohashi et al., 2007; Bouyer et al., 2008). For example, R2R3-MYB, bHLH, WD40, and HD-ZIP IV TFs appear to be essential for trichome development. Analysis of these important transcriptional regulators involved in the development of epidermal hair of *Arabidopsis* and fiber of cotton could contribute to systematically understand the molecular mechanisms of trichome development.

### V-myb Myeloblastosis Viral Oncogene Homolog TFs

MYB transcription factor constitutes a large and functionally diverse plant transcription factor family with a major role in plant-specific processes including biotic and abiotic stress responses (Mengiste et al., 2003), cell fate and identity determination (Kang et al., 2009), growth and developmental processes (Millar and Gubler, 2005), general and specialized metabolic responses (Zhou et al., 2009). [DE]Lx_2_[RK]x_3_Lx_6_Lx_3_R is a conserved motif in MYB proteins that is critical for interaction with *GLABRA3* (*GL3*; Zimmermann et al., 2004). In *Arabidopsis*, *GL1* encodes an R2R3-MYB TF, a regulator that functions at the earliest known stage of trichome initiation. Mutations in the *GL1* gene cause glabrous leaves (Oppenheimer et al., 1991). MYB23 is functionally equivalent to GL1, and they redundantly control trichome initiation at leaf edges (Kirik et al., 2005). MYB82 driven by the promoter of *GL1* was able to rescue the *gl1* mutant glabrous phenotypes, revealing that the MYB82 protein is also functionally similar to the GL1 protein (Liang et al., 2014). *MYB5* regulates trichome elongation and branching, and minimal changes in trichome morphology have been noted in the *myb5* mutant; however, the *myb5*/*myb23* double mutant produces a higher number of small and two-branched trichomes than the single mutant. Thus, *MYB5* and *MYB23* regulate trichome branching and extension in a partially redundant manner (Li et al., 2009). MYB106/NOK is a MIXTA-like TF that negatively regulates trichome branching in *Arabidopsis* (Jakoby et al., 2008). A group of single repeat R3-MYB TFs, including TRIPTYCHON (TRY; Schellmann et al., 2002), CAPRICE (CPC; Schellmann et al., 2002), TRICHOMELESS (TCL) 1 and 2 (Wang et al., 2007; Gan et al., 2011), and ENHANCER OF TRY AND CPC (ETC) 1–3 (Kirik et al., 2004; Wester et al., 2009) are negative regulators of trichome formation. For example, TCL1 negatively regulates trichome initiation, and over-expression of *TCL1* directly suppresses *GL1* transcription (Wang et al., 2007).

In cotton, some MYB TFs have evolved special roles in fiber development. GhMYB2 is a key regulator of fiber development in cotton and is homologous to AtGL1, which functions in trichome initiation in *Arabidopsis* (Guan et al., 2014a). Ectopic expression of *Gossypium arboreum MYB2* and *GhMYB2A* can complement the *gl1* mutant phenotype in *Arabidopsis* (Wang et al., 2004; Guan et al., 2014a). GhMYB5 exhibits a high similarity to AtMYB5 and is related to fiber initiation and elongation (Wang et al., 2021c). Sequence variations in the *cis*-elements of *Gossypium barbadense MYB5* and *GhMYB5* lead to differences in gene expression that are associated with natural variation in fiber development (Wang et al., 2021c). *GhMYB109* plays key role in cotton fiber development; *GhMYB109* knockdowns show a significant reduction in fiber length, unveiling a largely conserved role of R2R3-MYB genes in the cell fate determination (Pu et al., 2008). Fibers are elongated using sucrose as a direct carbon source. GhMYB212 directly controls expression of *GhSWEET12*, a sucrose transporter gene, in expanding fibers and is therefore required for cotton fiber elongation (Sun et al., 2019). MYBMIXTA-like TFs (MMLs) belong to the special subgroup 9 of R2R3-MYB proteins (Brockington et al., 2012; Hu et al., 2016). GhMML7 (GhMYB25) and GhMML3 (GhMYB25-like) proteins have been identified as regulators of fiber initiation and elongation. *GhMML7*-silenced cotton plants show a significant reduction in fiber length and leaf trichome numbers (Machado et al., 2009), whereas silencing *GhMML3* has no significant influence on trichome development (Walford et al., 2011). GhJAZ2, a repressor of JA signaling, negatively regulates fiber initiation by interacting with GhMML3 (Hu et al., 2016). GhMML4 is a sister MYB TF to GhMML3 that controls lint fiber development. Additionally, both of these two genes are tandemly arranged on the D12 chromosome (Wu et al., 2018). Cellulose contents during secondary cell wall (SCW) deposition phase of mature cotton fiber reach up to 90% (Han et al., 2013; Huang et al., 2021), and GhMYB7 directly regulates fiber cellulose synthesis by binding to three different *cis*-elements in the *GhCesA4*, *GhCesA7*, and *GhCesA8* promoters (Huang et al., 2021). Over-expression of *GhMYB7* significantly accelerates cellulose biosynthesis in the SCW, resulting in shorter fibers with thicker walls.

### Basic Helix–Loop–Helix TFs

In *Arabidopsis*, GL3 and ENHANCER OF GLABRA3 (EGL3) are functionally redundant bHLH TFs (Payne et al., 2000). Although mutation of *gl3* modestly affects trichome number and branching, *egl3* mutants have no significant trichome defects (Morohashi et al., 2007). Notably, *gl3/egl3* mutants have a completely glabrous phenotype (Zhang et al., 2003). Trichome initiation is co-regulated by *GL1* together with *GL3/EGL3* (Schiefelbein, 2003). *GL3* and *EGL3* are upregulated during trichome initialization and in young trichomes, then expression decreases in mature trichomes.

In cotton, GhDEL65 (a functional homologue of *Arabidopsis* GL3 and EGL3) regulates early fiber development (Shangguan et al., 2016). Ectopic expression of *GhDEL65* in the *Arabidopsis gl3/egl3* double mutant partly rescues the trichome-less phenotype, and over-expressing *GhDEL65* in wild-type *Arabidopsis* plants results in increased trichome density (Shangguan et al., 2016). *GhFP1* encodes a bHLH protein that positively regulates fiber elongation (Liu et al., 2020). GhFP1 directly binds to the *GhDWF4* and *GhCPD* promoters to activate brassinosteroid (BR) biosynthesis and signaling, as well as appropriate concentration of BR promotes cotton fiber elongation (Sun et al., 2005). Over-expression of *GhFP1* promotes trichome development in *Arabidopsis* (Liu et al., 2020). *PACLOBUTRAZOL RESISTANCE 1* (*PRE1*) is specifically expressed in fiber cells, and core *cis*-element variation in *GhPRE1* contributes to fiber formation (Zhao et al., 2018).

### WD40-Repeat TFs

The *Arabidopsis* protein TRANSPARENT TESTA GLABRA1 (TTG1) has four WD40 repeats and regulates trichome differentiation; loss of TTG1 function results in a glabrous phenotype (Walker et al., 1999). TTG1 physically binds with GL3 and forms a complex to control trichome initiation (Payne et al., 2000).

In cotton, *GhTTG1* and *GhTTG3* are functional homologues of *AtTTG1* (Humphries et al., 2005). Expression of either gene in *Arabidopsis ttg1* mutants rescues trichome development. GhWDR is a novel WD40-repeat protein. The number of WD40-repeat domains in GhWDR differs from those of AtTTG1 and GhTTG1-GhTTG4, implying functional differentiation (Tian et al., 2020). *GhWDR* is expressed in the entire process of fiber development, suggesting key contributions (Tian et al., 2020).

### Homeodomain-Leucine Zipper TFs

**Figure 3:**
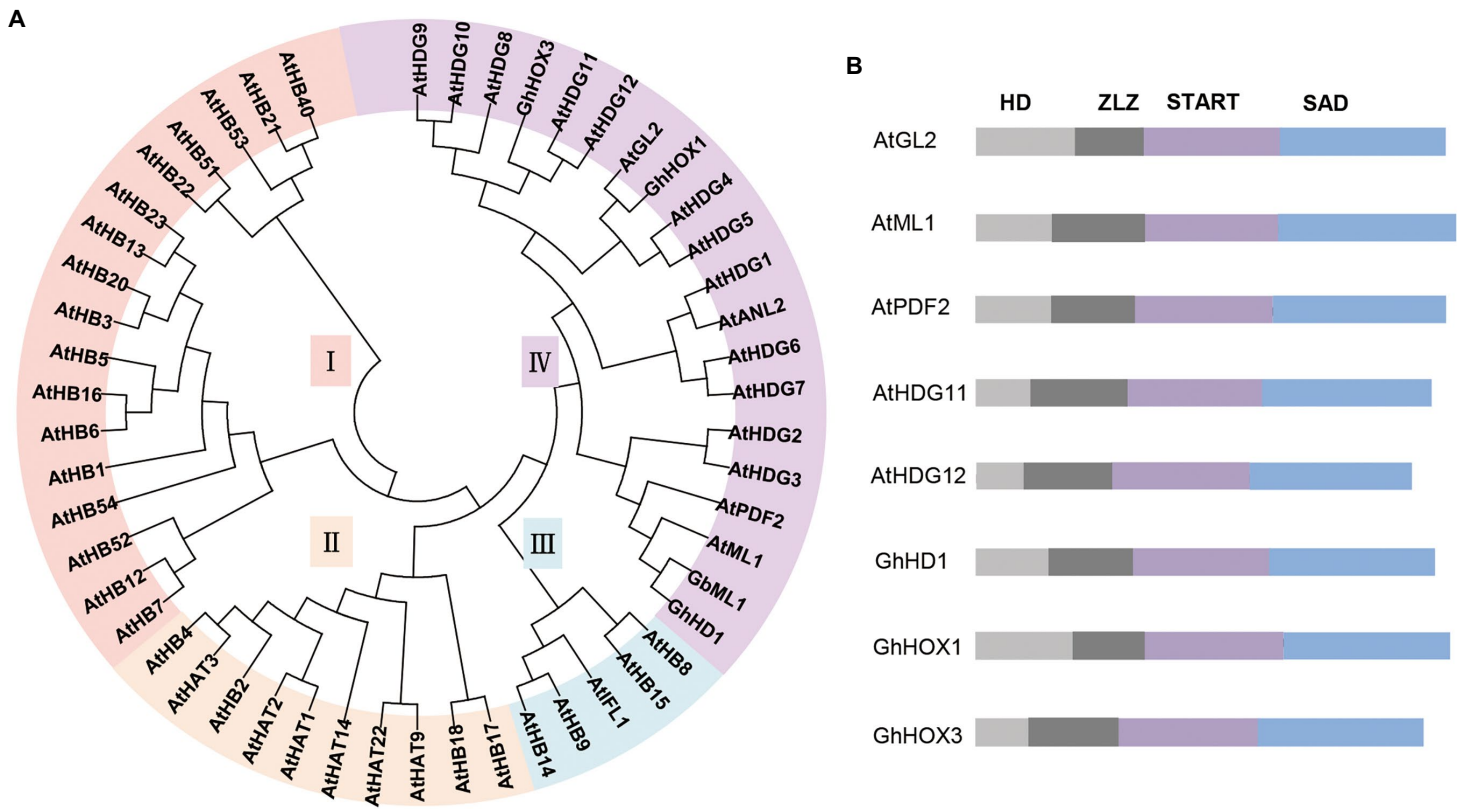
Relationships of HD-ZIP TF members in *Arabidopsis* and cotton. **(A)** Phylogenetic tree of HD-ZIP TFs in *Arabidopsis* and cotton by the neighbor-end joining method using MEGA 10.0. **(B)** Gene structure of the HD-ZIP IV TFs in *Arabidopsis* and cotton. ATHB1/HAT5 (At3g01470), ATHB3/HAT7 (At5g15150), ATHB5 (At5g65310), ATHB6 (At2g22430), ATHB7 (At2g46680), ATHB12 (At3g61890), ATHB13 (At1g69780), ATHB16 (At4g40060), ATHB20 (At3g01220), ATHB21 (At2g18550), ATHB22 (At2g36610), ATHB23 (At1g26960), ATHB40 (At4g36740), ATHB51 (At5g03790), ATHB52 (At5g53980), ATHB53 (At5g66700), ATHB54 (At1g27050), ATHB2/HAT4 (At4g16780), ATHB4 (At2g44910), ATHB17 (At2g01430), ATHB18 (At1g70920), HAT1 (At4g17460), HAT2 (At5g47370), HAT3 (At3g60390), HAT9 (At2g22800), HAT14 (At5g06710), HAT22 (At4g37790), ATHB8 (At4g32880), PHAVOLUTA/ATHB9 (At1g30490), PHABULOSA/ATHB14 (At2g34710), CORONA/ATHB15 (At1g52150), REVOLUTA/IFL1 (At5g60690), AtGL2 (At1g79840), AtML1 (At4g21750), AtPDF2 (At4g04890), AtANL2 (At4g00730), AtHDG1 (At3g61150), AtHDG2 (At1g05230), AtHDG3 (At2g32370), AtHDG4 (At4g17710), AtHDG5 (At5g46880), AtHDG6 (At4g25530), AtHDG7 (At5g52170), AtHDG8 (At3g03260), AtHDG9 (At5g17320), AtHDG10 (At1g34650), AtHDG11 (At1g73360), AtHDG12 (At1g17920), GhHD1 (Gh_A06G1283), GhHOX1 (AF530913), GhHOX3 (Gh_A12G2462).

A highly conserved DNA-binding homeodomain (HD) and leucine zipper (ZIP) motif characterize the HD-ZIP proteins, which constitute one of the largest plant-specific TF families (Tang et al., 2019). The ZIP motif mediates homodimerization and heterodimerization (Henriksson et al., 2005). In *Arabidopsis*, HD-ZIPs have been grouped into four major classes (I-IV) based on the exon-intron structures, similarity of nucleotide sequence, and the presence of additional sequences. (Henriksson et al., 2005; Perotti et al., 2019; Tang et al., 2019). HD-ZIP IV proteins have StAR-related lipid-transfer (START) and StAR-associated conserved (SAD) domains (Schrick et al., 2004; Figure 3B). HD-ZIP IV genes often exhibit predominant expression in a single tissue layer, typically limited to the epidermis and occasionally the subepidermal cell layer and they are closely related to trichome development (Abe et al., 2003; Gao et al., 2015). *GL2* was identified as the first identified HD-ZIP IV gene and is necessary for initiation and maintenance of trichomes (Marks et al., 2009). In *gl2* plants, trichome morphology is variable and expansion is aberrant (Szymanski et al., 1998). *GL2* has been shown to have functional redundancy with *HDG11* (Khosla et al., 2014). *hdg11* mutants have an excessively branched trichome phenotype (Nakamura et al., 2006). The HD-ZIP IV subfamily also contains ARABIDOPSIS THALIANA MERISTEM LAYER1 (ATML1) and its paralogue PROTODERMAL FACTOR2 (PDF2). ATML1 and PDF2 regulate the expression of meristem layer 1 (L1)-specific genes in epidermal cells, and *atml1*/*pdf2* double mutants have serious defects in shoot epidermal cell differentiation (Takada et al., 2013; Ogawa et al., 2015). GA is known to induce degradation of the REPRESSOR OF *ga1-3* (RGA) protein and activate the MBW complex to promote *GL2* expression, positively regulating trichome development (Qi et al., 2014). DELLA proteins also directly interact with ATML1 and PDF2, leading to the inhibition of L1-box gene expression (Rombolá-Caldentey et al., 2014).

In cotton, the *GL2* homologs *MERISTEM LAYER 1* (*GbML1*), *GhHD1*, *GaHOX1*, and *GhHOX3* are highly expressed in trichomes (Guan et al., 2008; Zhang et al., 2010; Walford et al., 2012; Shan et al., 2014). These cotton genes modify trichome development when ectopically expressed in *Arabidopsis*. *GbML1* controls cotton fiber development and interacts with a key regulator, GbMYB25 (Zhang et al., 2010). *GbML1* over-expression in *Arabidopsis* increases leaf and stem trichome density (Zhang et al., 2010). *GhHD1* is an L1-specific gene that regulates cotton epidermal cell differentiation (Walford et al., 2012). Knockout and over-expression experiments suggest that *GhHD1* has positive roles in trichome initiation (Walford et al., 2012). Recently, *GaHD1* was identified in the glabrous and fibreless cotton mutant line SMA-4; *GaHD1* is a candidate gene for trichome and fiber initiation (Ding et al., 2020). *GaHOX1* expression under the control of the *GL2* promoter could rescue the abnormal trichome phenotype of the *Arabidopsis* glabrous mutant *gl2-2* (Guan et al., 2008). Phylogenetic analysis shows that GhHOX1 is most closely related to AtGL2, whereas GhHD1 is most closely related to AtML1 and AtPDF2 (Figure 3A). Furthermore, over-expressing *GhHOX3* significantly increases fiber length, but *GhHOX3* knockdowns show decreased stem trichome density and fiber length, suggesting a vital role of *GhHOX3* in fiber elongation (Shan et al., 2014). Interestingly, GhHOX3 interacts with *GhHD1*, enhancing transcriptional activity of GhHOX3 (Shan et al., 2014). These results indicate that HD-ZIP IV proteins play crucial roles in the molecular regulation of cotton trichome and fiber development.

### The MBW Regulatory Complex

WD40-repeat proteins provide a scaffold in protein–protein interactions between R2R3-MYB and bHLH proteins. In *Arabidopsis*, GL1, GL3/EGL3, and TTG1 form a complex that induces trichome initiation by activating *GL2* expression (Arteaga et al., 2021). Single-repeat R3 MYB proteins (single-repeat MYBs) play key roles in controlling the trichome patterning in *Arabidopsis*. It was suggested that single-repeat MYBs compete with GL1 in binding to GL3/EGL3, thereby preventing the formation of activator complex GL1-GL3/EGL3-TTG1. Significantly, GL1-GL3/EGL3-TTG1 is needed for the activation of *GL2*, which is a positive regulator of trichome development (Ishida et al., 2008). In addition, GA induces RGA protein degradation, activating the GL1-GL3/EGL3-TTG1 complex and ultimately promoting *GL2* expression (Qi et al., 2014).

Protein–protein interaction analysis revealed that conserved amino acid signature ([DE]Lx2[RK]x3Lx6Lx3R) of MYB protein family is the structural basis of interaction between MYB and R/B-like BHLH proteins (Zimmermann et al., 2004). In cotton, MMLs comprise a specific family that regulates fiber development. Although GhMML4_D12 lacks the ([DE]Lx2[RK]x3Lx6Lx3R) motif, preventing interaction with bHLH proteins (Tian et al., 2020), it regulates lint fiber development by interacting with GhWDR, which is similar to the GL1-GL3/EGL3-TTG1 complex involved in *Arabidopsis* trichome development (Tian et al., 2020). Thus, cotton has likely evolved a specific regulatory network for fiber development.

### Other TFs

In *Arabidopsis*, other TFs have also been identified that control trichome development. C2H2 zinc finger proteins, including GLABROUS INFLORESCENCE STEMS (GIS), GIS2, GIS3, ZINC FINGER PROTEIN5 (ZFP5), ZFP6, and ZFP8 are key factors that regulate trichome initiation through GA and CK signaling (Gan et al., 2006, 2007; Zhou et al., 2011, 2013). *GIS3* is located upstream of *GIS*, *GIS2*, *ZFP8*, *GL1*, and *GL3*; *GIS* and *GIS2* are direct targets of *GIS3* (Sun et al., 2015). The TEOSINTE BRANCHED/CYCLOIDEA/PCF (TCP) class II protein TCP4 suppresses trichome branching by direct transcriptional activation of *GIS* (Vadde et al., 2018). A membrane-associated NAC (NAM, ATAF1/2, and CUC) TF, NTM1-LIKE8 (NTL8), is a regulator that acts upstream of *TRY* and *TCL1* in trichome initiation (Tian et al., 2017). *TTG2* encodes a WRKY protein that is a direct target of GL1 and has functional redundancy with *GL2* in regulating trichome development (Johnson et al., 2002; Ishida et al., 2007).

In cotton, in addition to the core regulatory factors described above, WRKY, TCP, and NAC TFs also play important roles in regulating fiber development. GhWRKY16 plays a crucial role in fiber initiation and elongation and is phosphorylated by GhMPK3-1 to directly upregulate downstream genes involved in early fiber development (Wang et al., 2021b). The functions of several TCP genes have been characterized with respect to fiber development. *GhTCP14* is a class I TCP gene that participates in fiber initiation and elongation and responds to exogenous auxin (Wang et al., 2013). *Arabidopsis GhTCP14* over-expressors have enhanced trichome and root hair development. A class II TCP protein, GhTCP4, maintains the balance between cotton fiber cell elongation and cell wall synthesis by interacting with GhHOX3 (Cao et al., 2020). Over-expression of *GhTCP4* accelerates biosynthesis of the SCW in fiber cells, resulting in shorter fibers and thicker walls. The NAC TF GhFSN1 is a positive regulator of fiber SCW biosynthesis, and over-expression increases wall thickness and slightly decreases fiber length (Zhang et al., 2018).

## Chromatin-Mediated Regulation of Trichome Development

Chromatin stability and dynamics are important in regulation of gene expression (Hung et al., 2020). Changes in chromatin structure are closely associated with DNA methylation, histone modifications, and DNA binding to histones (Peterson and Laniel, 2004). DNA methylation status affects the binding ability of proteins to chromatin. Histone modifications include acetylation (ac), methylation (me), ubiquitination (ub), and phosphorylation. Modifications such as H3K9ac and H3K4 trimethylation (H3K4me3) are generally associated with active transcription, whereas H3K9me2 and H3K27me3 are involved in transcriptional inhibition (Zheng et al., 2016). The chromatin assembly factor CAF-1 is a histone chaperone that promotes chromatin formation and maintains specific chromatin states (Exner et al., 2008). *CAF-1* is required for *Arabidopsis* trichome branching in an endoreduplication-independent pathway (Exner et al., 2008).

### Histone Modifications

**Figure 4:**
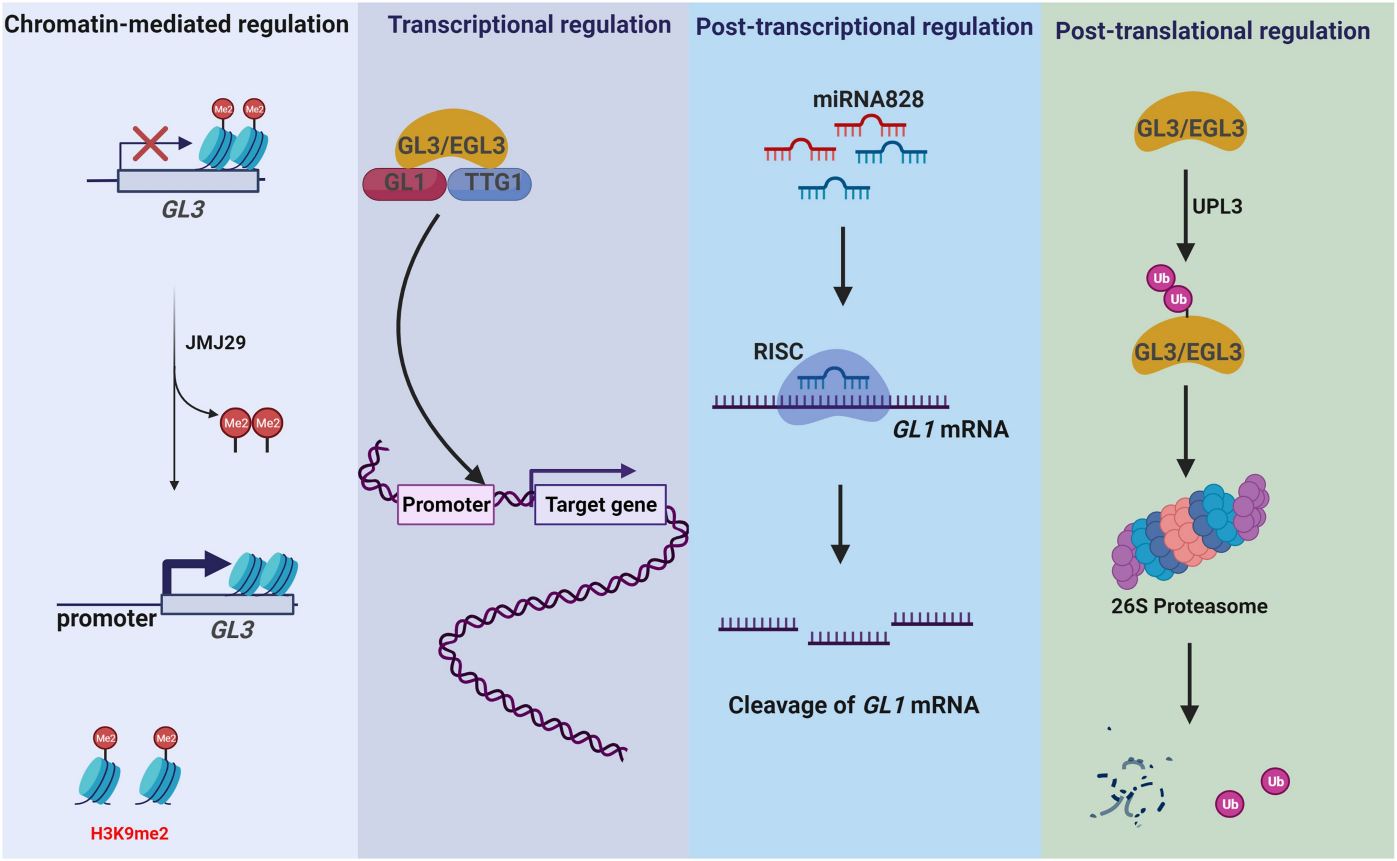
*GL3/EGL3* and *GL1* are involved in multi-dimensional regulation of *Arabidopsis* trichrome development. GL3/EGL3 and GL1 are important regulators of *Arabidopsis* trichome development. They control trichome development by different regulation modes. JMJ29 directly demethylates H3K9 on the *GL3* locus, thereby regulating *GL3* expression in trichome initiation (chromatin-mediated regulation). As TFs, GL3/EGL3 and GL1 regulate the expression of trichome development-related genes (transcriptional regulation). In *Arabidopsis*, miR828 promotes the degradation of *GL1* and inhibits trichome formation (post-transcriptional regulation). In addition, ubiquitination modification controls the level of GL3. UPL3 mediates the proteasome degradation of GL3/EGL3 and represses the formation of GL1-GL3/EGL3-TTG1 complex (post-translational regulation).

Histone acetylation is dynamically maintained by histone acetyltransferases and histone deacetylases (HDACs). In *Arabidopsis*, the histone acetyltransferase GCN5 is a member of the Spt-Ada-Gcn5 acetyltransferase (SAGA) complex (Wu et al., 2021). GCN5 regulates expression of genes involved in trichome development by acetylating histone H3 (Wu et al., 2021) and regulates histone acetylation of promoters in *GL1*, *GL2*, *GL3*, and *CPC*, which are involved in trichome initiation (Wang et al., 2019a). Moreover, GCN5 is required for trichome branching, as demonstrated by the significantly less branched phenotype of the *gcn5-1* mutant (Kotak et al., 2018). Histone methylation, regulated by methyltransferases and demethylases, results in gene activation or repression by affecting the chromatin state (Hung et al., 2020). JMJ29 is a histone demethylase that contains a JmjC domain and is responsible for demethylation at H3K9me1/2 in *Arabidopsis* (Hung et al., 2020). JMJ29 directly demethylates H3K9 on the *GL3* locus, thereby regulating *GL3* expression in trichome initiation (Figure 4).

In cotton, HDAC activity is essential for fiber initiation, and GhHDA5 primarily deacetylates H3K9ac marks (Kumar et al., 2018). *GhHDA5* knockdowns show significantly suppressed fiber initiation and yield. *GhH2A12* is a histone H2A gene that controls fiber initiation and early elongation by regulating cell cycle-related genes (Hao et al., 2014). *GhH2A12* over-expression delays fiber initiation and results in shorter fibers. These results provide deeper insights into the molecular mechanisms of chromatin-mediated regulation of trichome development.

### DNA Methylation

In plants, DNA methylation occurs at CG, CHG, and CHH sites through distinct pathways (Song et al., 2015). In cotton, CHH methylation is distinctly changed during ovule and fiber development, and CHG and CHH methylation contribute to homoeologous gene expression bias in ovules and fibers (Song et al., 2015). Moreover, heterochromatic DNA hypermethylation affects *G. barbadense* fiber differentiation through an H3K9me2-dependent pathway (Wang et al., 2016).

## Post-Transcriptional Regulation of Trichome Development

Gene expression regulation at the post-transcriptional level is crucial for plant growth and development. Non-coding RNA (ncRNA) regulation and mRNA modification have emerged as important components of post-transcriptional gene expression. ncRNAs include long noncoding RNAs (lncRNAs), small interfering RNAs (siRNAs), and microRNAs (miRNAs). lncRNAs are defined as ncRNAs with transcripts longer than 200 nt; some lncRNAs are known to manipulate fiber initiation (Hu et al., 2018). miRNAs are small single-stranded ncRNAs that are 20–22 nt in length and have important roles in modulating post-transcriptional gene expression in plants (Voinnet, 2009; Pattanaik et al., 2014). N6-methyladenosine (m^6^A) controls mRNA fate and expression and is the most abundant and widespread internal mRNA modification. m^6^A can be dynamically read, written, and erased (Wei et al., 2018). RNA-binding proteins with a YTH domain act as anchors for m^6^A readers.

### Non-coding RNA Regulation

Recent studies have identified miRNAs that are involved in regulating trichome development in *Arabidopsis* (Figure 2), such as miR156 (Yu et al., 2010), miR171 (Xue et al., 2014), miR172 (Lian et al., 2021), and miR319 (Vadde et al., 2018). For example, miR156 targets *SQUAMOSA PROMOTER BINDING PROTEIN LIKE9* (*SPL9*), which represses trichome formation on the inflorescence, to regulate trichome development in *Arabidopsis* (Yu et al., 2010). Overexpressing miR156 can cause ectopic trichome on the stem and floral, while plants with improved transcripts level of *SPL9* significantly decrease of trichome density. Furthermore, *TCL1* and *TRY*, which are negative regulators of trichome development, are target genes of SPL9. *LOST MERISTEMS* (*LOM*) *1*, *LOM2*, and *LOM3* are targeted by miR171; *miR171* over-expression decreases trichome density on *Arabidopsis* stem and floral organs (Xue et al., 2014). Interestingly, LOM1-3 are involved in modulating *SPL9* activity. miR156 and miR171 form a regulatory network through direct interaction of their target proteins. miR172 family members have different expression patterns and functional specificity, and elevated levels of miR172 promote trichome formation on the abaxial leaf surfaces (Lian et al., 2021). A recent study provided insight into the coordinated regulation of trichome initiation in *Paulownia tomentosa* by miR319/TCP19 and GA signaling (Fan et al., 2020), showing that *miR319* over-expression significantly elevated trichome density. In *Arabidopsis*, *TCP4* is targeted by miR319 and suppresses trichome branching (Vadde et al., 2018).

Several studies have identified ncRNAs that are expressed during cotton fiber development (Guan et al., 2014b; Wang et al., 2015; Wan et al., 2016). Through genome and RNA sequencing, lncRNAs in *G. barbadense* have been shown to exhibit homoeologous expression bias (Wang et al., 2015). GhMML3-derived endogenous siRNA involved in regulation of fiber cell development by mediating the self-cleavage of GhMML3 transcript and subsequently result in the development of naked seeds (Wan et al., 2016). In cotton, miR828 and miR858 have been shown to control fiber development by targeting *GhMYB2* homologs. Another report demonstrated that miRNA156/157 is essential for fiber elongation in *G. barbadense* (Liu et al., 2014).

### mRNA Modifications

In *Arabidopsis*, 11 YTH proteins have been identified that have a highly conserved C-terminal region, EVOLUTIONARILY CONSERVED C-TERMINAL REGION (ECT) 1–11 (Ok et al., 2005). ECT2 is an m^6^A reader that controls normal trichome morphology in *Arabidopsis* (Wei et al., 2018; Figure 2); its deletion leads to defects in trichome branching (Scutenaire et al., 2018). ECT2 can bind to the promoters of *TTG1*, *DISTORTED TRICHOME 2* (*DIS2*), and *IRREGULAR TRICHOME BRANCH 1* (*ITB1*), which are m^6^A-modified genes that regulate trichome development (Liang et al., 2020). In *ect2* mutants, *TTG1*, *ITB1*, and *DIS2* transcripts are degraded, which affects trichome branching (Wei et al., 2018). These results demonstrate molecular mechanisms by which m^6^A mediates trichome development through recruiting reader proteins.

## Post-Translational Regulation of Trichome Development

Great progress has been made in understanding the regulatory mechanism of the MBW complex in trichome development, but it remains important to clarify the factors that regulate the transcriptional activator complex at the post-translational dimension. Post-translational modifications include (but not limited to), acetylation, glycosylation, ubiquitination, phosphorylation, glycation, SUMOylation, methylation, nitration, and oxidation. The ubiquitin/26S proteasome system (UPS) is an important regulator and mediates the proteasomal degradation of TFs (Patra et al., 2013). The UPS consists of the enzymes E1, E2, and E3; E3 is the key factor that determines substrate specificity (Vierstra, 2009). Moreover, histone H2B is ubiquitinated by E2 enzymes (UBC1, UBC2, and UBC3) and E3 ligases (HUB1 and HUB2), and H2B mono-ubiquitination (H2Bub1) triggers plant growth (Fleury et al., 2007; Feng et al., 2018).

In *Arabidopsis*, *KAKTUS* (*KAK*) encodes a HECT-type E3 ubiquitin ligase that negatively regulates endoreduplication cycles in trichome branching by accelerating GL3 and EGL3 degradation (El Refy et al., 2004). Genetic studies have revealed a specific role of ubiquitin protein ligase 3 (UPL3) in trichome development (Downes et al., 2003; Patra et al., 2013), and proteasomal degradation of GL3 and EGL3 is mediated by UPL3 (Figure 4). In addition, *UPL3* is downregulated in *gl3* mutants. As a subunit of the 26S proteasome, REGULATORY PARTICLE AAA-ATPASE 2a (RPT2a) also controls trichome branching by negatively regulating endoreduplication (Sako et al., 2010). Mutation of *rpt2a* suppresses the *gl3* phenotype, but *rpt2a*/*gl3* double mutants have normal trichome branching. These findings highlight the importance of post-translational regulation in trichome development.

GhHUB2 is a cotton ubiquitin ligase involved in fiber elongation and SCW deposition (Feng et al., 2018). GhHUB2 interacts with a fiber transcriptional repressor, GhKNL1, and degrades GhKNL1 through the ubiquitin-26S proteasome pathway. Furthermore, protein phosphorylation and acetylation have been reported to be involved in fiber development (Ma et al., 2014; Singh et al., 2020). These reports provide new insights for further study into the mechanisms of fiber development in cotton.

## Conclusion and Perspective

Trichome development is a highly complex process, involving the coordinated function of many genes and signaling pathways to integrate a variety of exogenous and endogenous factors. Previous studies have supported a model of trichome development as controlled by a multi-dimensional regulatory network, including chromatin-mediated, transcriptional, post-transcriptional, and post-translational regulation. Elucidation of molecular and genetic regulatory mechanisms of trichome development is ongoing, and further insight is necessary to understand trichome development from the perspective of multi-dimensional network modules and their interactions. For example, some genes have been shown to be involved in *Arabidopsis* trichome development at multiple regulatory levels. The TF GL3 is involved in trichome initiation and branching and is regulated by JMJ29 demethylase through demethylation of H3K9me2 (Hung et al., 2020). GL3 also forms a core complex of trichome initiation with GL1 and TTG1 to regulate the expression of *GL2*/*TTG2* (Wang et al., 2021a); furthermore, it can be ubiquitinated by UPL3, resulting in degradation of the GL1-GL3/EGL3-TTG1 complex (Patra et al., 2013).

Comparative studies into the molecular mechanisms in *Arabidopsis* trichomes and cotton fibers will accelerate the understanding of trichome development in different plant species. Cotton fiber development is a longer process than that of *Arabidopsis* trichomes and involves a more complex regulatory network. However, the mechanism of *Arabidopsis* trichome development provides a molecular basis for understanding cotton fiber development; ectopic expression of genes regulating cotton fiber initiation and elongation significantly affect trichome phenotypes in *Arabidopsis*, reflecting functional homology between the species (Table 1). However, the regulatory networks of cotton fiber development are distinct from those of *Arabidopsis* trichomes. For example, the MML4-WDR complex is present in cotton in place of the *Arabidopsis* GL1-GL3/EGL3-TTG1 complex (Tian and Zhang, 2021). In addition to the mechanisms reviewed here, fiber development also includes metabolic regulatory pathways, such as sucrose metabolism (Ding et al., 2021). Fiber elongation phase utilizes sucrose as a direct carbon source during cellulose biosynthesis and also provides turgor pressure to accelerate fiber elongation. However, molecular mechanism of transcriptional regulation of sucrose transportation from ovules into elongating fibers remains elusive.

After the release of recent updated cotton reference genome (Ma et al., 2021), the identification of the molecular regulatory mechanisms of fiber development will be accelerated. Previous reports have shown that some homologous genes are differentially expressed in allotetraploid cotton, and homoeolog bias is responsible for ovule and fiber development (Zhao et al., 2018; Ando et al., 2021). Moreover, epigenetic modifications, such as histone modification and DNA methylation, are important factors influencing homoeology bias (Zheng et al., 2016; Kumar et al., 2018). Large number of homoeologous genes with expression bias designates that they have been profoundly sub-functionalized for cotton fiber development. These findings will promote the study of functional differences in homoeologs.

In the future, additional studies should be conducted to study trichome optimization. The rapid development and effective application of CRISPR gene editing techniques in plants will allow for easier generation of gene knockins and knockouts (Miki et al., 2018; Lu et al., 2020) compared to older techniques such as cross-breeding to produce target phenotypes. In higher plants, knockdown of negative regulators by gene editing is expected to confer positive, beneficial phenotypes. For example, glume trichomes affect rice quality. Conversely, for positive regulatory factors, economic benefits could be greatly improved by knocking in genes, such as those associated with increased cotton fiber yield.

## Author Contributions

RZ and CL conceived the concept of the review. YaW, QZ, ZM, YWe, MA, and YuW compiled the literature. YaW and CL designed the figures. YaW, RZ, and CL wrote the paper. All authors contributed to the article and approved the submitted version.

## Funding

We regret that many original articles on plant epidermal hairs development and cotton fiber development. CL and RZ were supported by the National Natural Science Foundation of China (32072115 and 31771850), the National Special Program for GMO Development of China (No. 2016ZX08009003-003-004 and 2016ZX08005-004), and the Agricultural Science and Technology Innovation Program of Chinese Academy of Agricultural Sciences.

## Conflict of Interest

The authors declare that the research was conducted in the absence of any commercial or financial relationships that could be construed as a potential conflict of interest.

## Publisher’s Note

All claims expressed in this article are solely those of the authors and do not necessarily represent those of their affiliated organizations, or those of the publisher, the editors and the reviewers. Any product that may be evaluated in this article, or claim that may be made by its manufacturer, is not guaranteed or endorsed by the publisher.
